# Edoxaban in the Evolving Scenario of Non Vitamin K Antagonist Oral Anticoagulants Imputed Placebo Analysis and Multiple Treatment Comparisons

**DOI:** 10.1371/journal.pone.0100478

**Published:** 2014-06-23

**Authors:** Paolo Verdecchia, Fabio Angeli, Gregory Y. H. Lip, Gianpaolo Reboldi

**Affiliations:** 1 Department of Medicine, Hospital of Assisi, Assisi, Italy; 2 Cardiology and Cardiovascular Pathophysiology, University Hospital of Perugia, Perugia, Italy; 3 University of Birmingham Centre for Cardiovascular Sciences, City Hospital, Birmingham, United Kingdom; 4 Department of Medicine, University of Perugia, Perugia, Italy; Universidad Peruana de Ciencias Aplicadas (UPC), Peru

## Abstract

**Background:**

Edoxaban recently proved non-inferior to warfarin for prevention of thromboembolism in patients with non-valvular atrial fibrillation (AF). We conducted an imputed-placebo analysis with estimates of the proportion of warfarin effect preserved by each non vitamin K antagonist oral anticoagulant (NOAC) and indirect comparisons between edoxaban and different NOACs.

**Methods and Findings:**

We performed a literature search (up to January 2014), clinical trials registers, conference proceedings, and websites of regulatory agencies. We selected non-inferiority randomised controlled phase III trials of dabigatran, rivaroxaban, apixaban and edoxaban compared with adjusted-dose warfarin in non-valvular AF. Compared to imputed placebo, all NOACs reduced the risk of stroke (ORs between 0.24 and 0.42, all p<0.001) and all-cause mortality (ORs between 0.55 and 0.59, all p<0.05). Edoxaban 30 mg and 60 mg preserved 87% and 112%, respectively, of the protective effect of warfarin on stroke, and 133% and 121%, respectively, of the protective effect of warfarin on all-cause mortality. The risk of primary outcome (stroke/systemic embolism), all strokes and ischemic strokes was significantly higher with edoxaban 30 mg than dabigatran 150 mg and apixaban. There were no significant differences between edoxaban 60 mg and other NOACs for all efficacy outcomes except stroke, which was higher with edoxaban 60 mg than dabigatran 150 mg. The risk of major bleedings was lower with edoxaban 30 mg than any other NOAC, odds ratios (ORs) ranging between 0.45 and 0.67 (all p<0.001).

**Conclusions:**

This study suggests that all NOACs preserve a substantial or even larger proportion of the protective warfarin effect on stroke and all-cause mortality. Edoxaban 30 mg is associated with a definitely lower risk of major bleedings than other NOACs. This is counterbalanced by a lower efficacy in the prevention of thromboembolism, although with a final benefit on all-cause mortality.

## Introduction

Vitamin K antagonists (VKA) have long been the only oral anticoagulant agents available for effective thromboprophylaxis in patients with atrial fibrillation (AF). In a landmark meta-analysis of trials conducted in AF patients randomized to either adjusted-dose warfarin versus placebo or control for a mean exposure time of 1.6 years per patient, warfarin reduced the risk of stroke by 64% (95% confidence interval (CI): 49% to 74%), and that of ischemic stroke by 67% (CI: 54% to 77%), as well as a reduction in all-cause mortality by 26% (CI 3% to 43%) [1].

This impressive benefit made it unethical to compare any non vitamin K antagonist oral anticoagulant (NOAC) [2] with placebo in subsequent outcome trials. Consequently, the major studies published over the past few years with the direct thrombin inhibitor dabigatran [3] and the factor Xa inhibitors rivaroxaban [4], apixaban [5] and, lastly, edoxaban [6], were well-designed non-inferiority trials of each single NOAC versus adjusted-dose warfarin. Notably, any inference about the efficacy of NOACs from these studies assumes that the benefit of warfarin in preventing stroke and systemic embolism approaches that found in prior trials vs placebo or control, as summarized in the above mentioned meta-analysis [1].

After these studies, dabigatran, rivaroxaban and apixaban gained regulatory approval in many countries for prevention of stroke in patients with non valvular AF. The dose of dabigatran 110 mg b.i.d. has not been approved in the Unites States by the Food and Drug Administration (FDA), that approved the 75 mg b.i.d. dose in patients with glomerular filtration rate between 15 and 29 ml/min [7].

Although these drugs are valuable alternative to warfarin [8], [9], the physician has few arguments to direct his/her choice to one over the other in the absence of direct head-to-head comparisons. Several indirect comparisons have been conducted between dabigatran, rivaroxaban and apixaban [10]–[15]. In the context of limitations of indirect comparisons [16], [17], these analyses suggest a lower risk of stroke/systemic embolism with dabigatran 150 mg bid versus dabigatran 110 mg bid and rivaroxaban, and a lower risk of major bleedings with dabigatran 110 mg bid and apixaban versus dabigatran 150 mg bid and rivaroxaban [18], [19].

More recently, edoxaban emerged as the fourth NOAC in its class. In the *Effective Anticoagulation with Factor Xa Next Generation in Atrial Fibrillation–Thrombolysis in Myocardial Infarction 48* (ENGAGE AF-TIMI 48) trial, 21,105 patients with non valvular AF were randomized to adjusted-dose warfarin or two doses (30 mg q.d., 60 mg q.d.) of edoxaban [6]. The primary efficacy endpoint was a composite of stroke and systemic embolism and the main safety end-point was major bleeding [6]. Both doses of edoxaban were non inferior to warfarin for the prevention of stroke and systemic embolism [6]. Thus far, edoxaban has not yet gained approval by FDA and other regulatory Agencies.

The ENGAGE-AF trial [6] expanded the horizon of available alternatives to VKA and offered the opportunity of a more comprehensive evaluation of this class of drugs. In the light of this new trial, the present study has three goals: (1) to estimate the proportion of warfarin effect preserved by each of the NOACs and their efficacy versus a putative placebo on the risk of stroke and all-cause mortality; (2) to update the previous estimates of benefits and harms of NOACs as a whole versus warfarin; (3) to estimate, through indirect comparisons, the relative efficacy and safety of either dose of edoxaban versus different NOACs.

## Methods

### Study selection

**Figure 1:**
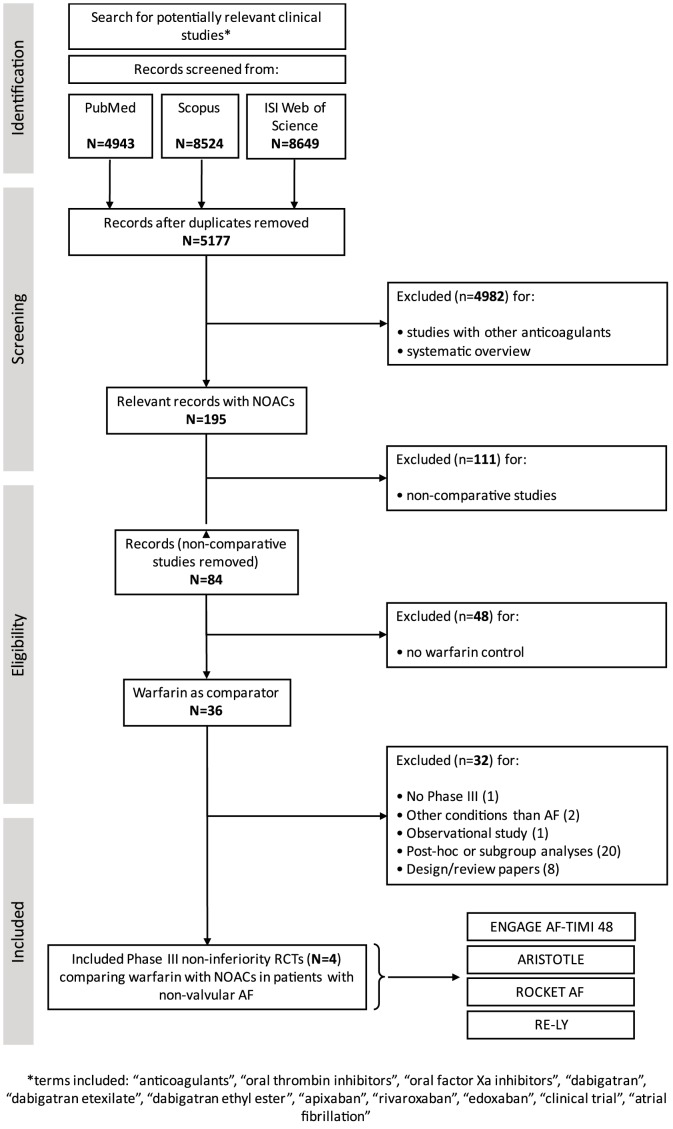
Search strategy and selection of clinical trial according to the PRISMA (Preferred Reporting Items for Systematic reviews and Meta-Analyses) statement for reporting systematic reviews and meta-analyses.

We used the PRISMA (Preferred Reporting Items for Systematic reviews and Meta-Analyses) statement for reporting systematic reviews and meta-analyses of randomized controlled trials (RCTs) as a guide for this study (PRISMA checklist S1) [20], including the preparation of a protocol and analysis plan (Protocol S2). Following a literature search (up to January 2014), to perform indirect comparisons and imputed placebo analyses in the non-inferiority setting, we identified four large phase III studies (Figure 1): *Randomized Evaluation of Long-Term Anticoagulation Therapy* (RE-LY) [3], *Rivaroxaban Once Daily Oral Direct Factor Xa Inhibition Compared with Vitamin K Antagonism for Prevention of Stroke and Embolism Trial in Atrial Fibrillation* (ROCKET-AF) [4], *Apixaban for Reduction in Stroke and Other Thromboembolic Events in Atrial Fibrillation* (ARISTOTLE) [5], and ENGAGE AF-TIMI 48 [6] (see table 1 for the complete summary of trials'characteristics). For RE-LY [3], we integrated the original data with the update published in 2010 [21]. As shown in figure 1, we excluded systematic overviews or studies with different anticoagulants (n = 4748), non comparative studies (n = 111), studies without warfarin control (n = 48) and other 32 studies for a variety of reasons reported in the table. We included only active control phase III non-inferiority studies because our aim was to provide estimates of the proportion of warfarin effect preserved by NOACs and their efficacy versus an imputed placebo as a measure of assay sensitivity. Active controlled trials might be uninformative as they can neither demonstrate the effectiveness of a new agent nor provide a valid comparison to control therapy unless assay sensitivity can be assured [22], [23].

**Table 1:** Main Characteristics of Trials Evaluating New Oral Anticoagulants for Stroke Prevention in Patients with Nonvalvular Atrial Fibrillation.

Characteristics	RE-LY Dabigatran	ROCKET AF Rivaroxaban	ARISTOTLE Apixaban	ENGAGE AF Edoxaban
Randomized patients, N	18,113	14,264	18,201	21,105
Countries	44 (951 Centers)	45 (1178 Centers)	39 (1034 Centers)	46 (1393 Centers)
Allocation	D 110 mg b.i.d.: N = 6,015	R 20 mg q.d.: N = 7,131 Warfarin: N = 7,133	A 5 mg q.d.: N = 9,120 Warfarin: N = 9,081	E 30 mg q.d.: N = 7,034
	D 150 mg b.i.d.: N = 6,076			E 60 mg q.d.: N = 7,035
	Warfarin: N = 6,022			Warfarin: N = 7,036
Study design	Open label vs. warfarin	Double-blind	Double-blind	Double-blind
	Double-blind D 150 vs. D 110			
Patients lost to follow-up	20	32	69	1
Median duration of follow-up, years	2.0	1.9	1.9	2.8
Age, years	71 (mean)	73 (median)	70 (median)	72 (median)
Female, N	6,599	5,663	6,416	8,040
CHADS_2_, mean	2.2	3.5	2.1	2.8
Creatinine clearance ≤50 ml/min	19.3	20.7	16.6	19.3
Paroxysmal AF, %	32.8	17.6	15.3	25.4
Prior stroke, TIA or systemic thromboembolism, %	20.0*	54.8	19.4	28.3*
Heart failure, %	32.0	62.5	35.4	57.4
Diabetes mellitus, %	23.3	40.0	25.0	36.1
Hypertension, %	78.9	90.5	87.5	93.5
Drugs at baseline				
Aspirin, %	39.8	36.5	30.9	29.2
Vitamin K antagonist, %	49.6	62.4	57.2	58.9
Average TTR in the warfarin group	64	55	62	65

D, Dabigatran; R, Rivaroxaban; A, Apixaban; E, Edoxaban; N, number of patients; AF, atrial fibrillation; TIA, transient ischemic attack, TTR = time in therapeutic range (International normalized ratio 2.0 to 3.0); CHF, congestive heart failure; DM, diabetes mellitus; HTN, hypertension; CHADS_2_ indicates CHF, hypertension, age, diabetes mellitus, stroke.

*  =  Stroke or TIA only.

We extracted data on both efficacy and safety outcomes as detailed below and in the study protocol (Protocol S2). Additional data on outcomes, not available in the main papers of included studies, were retrieved from the FDA website (http://www.fda.gov/Drugs/InformationOnDrugs/).

The authors of this study independently extracted all outcome data using a pre-specified form and disagreements were resolved through discussion.

### Data synthesis and statistical analysis

Efficacy outcomes included the composite of stroke and systemic embolism (i.e., the primary efficacy outcome event in each of the four trials [3]–[6]), stroke (i.e., all strokes), hemorrhagic stroke, ischemic or uncertain type of stroke and systemic embolism. For safety, we considered major bleeding, intracranial bleeding, gastrointestinal bleeding, myocardial infarction and all-cause death.

In keeping with previous studies [10], [18] the expected effect of NOACs as a class versus warfarin, was calculated as a weighted average using the inverse of the variance of the log(odds ratio (OR)) as weights. For this analysis, the higher doses of dabigatran (150 mg b.i.d. arm of RE-LY [3]) and edoxaban (60 mg q.d. arm of ENGAGE AF-TIMI 48 [6]) were analyzed with data from ROCKET-AF [4] and ARISTOTLE [5]. In a separate analysis, we analyzed the lower doses of dabigatran (110 mg b.i.d. arm of RE-LY [3]) and edoxaban (30 mg q.d. arm of ENGAGE AF-TIMI 48 [6]) with data from the other two trials.

We used the methodology introduced by Hasselblad and Kong to estimate the effects of NOACs versus imputed placebo [24]. Such approach assumes that previous trials tested warfarin versus placebo using the same outcome event as in the trials of NOACs versus warfarin, and that the populations exposed to trials of warfarin vs placebo and warfarin versus NOACs are similar [24]. The imputed placebo approach also relies on the assumption of “constancy” of the beneficial effect of warfarin versus placebo as observed in previous controlled trials [25]. This last assumption, however, is conditioned by the differences in patient characteristics, concomitant medications, intensity of treatment, and other trial design features [22], [24], [26], [27]. In addition, stroke rate seems to be declining over time both in the general population [28] and in AF patients treated with warfarin [29]. An effective way to “discount” for this limitation is to estimate the proportion of the warfarin treatment effect retained by each NOAC [25], [30], [31]. This is accomplished by determining the ratio of the effect of the new treatment versus putative placebo relative to the effect of the standard treatment versus placebo along with its estimated variance and CI [22], [24], [26], [27]. To prevent further limitations due to the use of a composite outcome (stroke and systemic embolism) in new trials as opposed to older ones, we restricted the imputed placebo analysis to stroke and all-cause mortality, as unequivocal and comparable outcome events in the trials of warfarin vs. placebo [1], [32] and NOACs versus warfarin [3]–[6]. For this purpose, the warfarin treatment effect was derived from a random-effects meta-analysis of 6 historical placebo-controlled trials [33]–[38] using the OR as the analysis metric.

We made multiple treatment comparisons between edoxaban and other NOACs using the Bucher method [39], [40] with warfarin used as common comparator. Because of the limited number of trials and in the absence of head-to-head comparisons between different NOACs, we did not make a formal network or mixed treatment comparison meta-analysis, in line with the recommendations and caveats outlined by the International Society for Pharmacoeconomics and Outcomes Research [41], [42]. In brief, we estimated the OR of an event with a given NOAC (NOAC_1_) versus another NOAC (NOAC_2_) (OR_NOAC1/NOAC2_) by dividing the OR of NOAC_1_ versus warfarin (OR_NOAC1/warfarin_) by the OR of NOAC_2_ versus warfarin (OR_NOAC2/warfarin_). We estimated the OR of selected events for each dose of edoxaban versus dabigatran (each dose), rivaroxaban and apixaban. The Bucher method assumes that the differences between a given NOAC and warfarin in terms of efficacy and safety would have been analogous if tested in different trial populations exposed to different NOACs versus warfarin [16]. However, since different studies were not fully comparable for some features including the thromboembolic risk, reflected by the CHADS_2_ score, the time in therapeutic range and other methodological aspects (open-label versus double-blind), indirect comparisons should be interpreted prudently [16], [17].

We used the R software version 3 (R Foundation for Statistical Computing, Vienna, Austria. URL http://www.R-project.org) for the analyses, with pre-specified efficacy and safety outcomes.

## Results

In aggregate, the four trials [3]–[6] accrued 71,683 patients. Table 1 shows the main features of the four studies [3]–[6]. The sample size was larger (N = 21,105), and the median duration of follow-up longer (2.8 years) in the ENGAGE AF-TIMI 48 [6] than in the other studies. Similar to ROCKET-AF [4] and ARISTOTLE [5], ENGAGE AF-TIMI 48 [6] was a double-blind trial vs warfarin [6], whereas RE-LY [3] was an open-label study of dabigatran versus warfarin with a double-blind comparison between the two different dabigatran doses [3]. Based on the CHADS_2_ score [43], the risk of stroke in the ENGAGE AF-TIMI 48 trial [6] was intermediate (2.8 points) between ROCKET-AF [4] (3.5 points) on a side, and RE-LY [3] (2.2 points) and ARISTOTLE [5] (2.1 points) on the other side. In terms of prevalence of heart failure, diabetes and hypertension at baseline, the ENGAGE AF-TIMI 48 [6]was more similar to ROCKET-AF [4] than to the other two studies. The average time in therapeutic range was 64.9% (median time 68%) in ENGAGE AF-TIMI 48 [6], as opposed to 64% in RE-LY [3], 55% in ROCKET AF [4] and 62% in ARISTOTLE [5].

### Weighted average effect versus warfarin

When the higher doses of dabigatran and edoxaban were used for the estimates versus warfarin (Table 2, left side), NOACs as a whole reduced the risk of stroke/systemic embolism (by 21%; p<0.001), stroke (by 20%; p<0.001), hemorrhagic stroke (by 50%; p<0.001) and systemic embolism (by 40%; p = 0.006). On the safety side, NOACs reduced the risk of major bleedings (by 15%; p<0.001), intracranial bleedings (by 52%; p<0.001) and death from any cause (by 10%; p = 0.001). Ischemic (or uncertain) stroke and myocardial infarction did not differ significantly between NOACs and warfarin, whereas gastrointestinal bleeding were more common with NOACS than with warfarin (by 29%; p<0.001).When using the lower doses of dabigatran and edoxaban (Table 2, right side), NOACs reduced stroke/systemic embolism (by 9%; p = 0.041) and hemorrhagic stroke (by 37%; p<0.001). All strokes (p = 0.166) and systemic embolism (p = 0.225) did not differ, while the ischemic (or uncertain) type of stroke was more frequent with NOACs than with warfarin (by 13%; p = 0.021). On the safety side, NOACs reduced the risk of major bleedings (by 27%; p<0.001), intracranial bleedings (by 59%; p<0.001) and all-cause death (by 91%; p<0.001), whereas gastrointestinal bleedings (p = 0.688) and myocardial infarction (p = 0.640) did not differ between NOACs and warfarin.

**Table 2.Weighted:** Average Effects of New Oral Anticoagulants Versus Warfarin.

	Any NOAC (Dabigatran 150 mg BID, Apixaban 5 mg BID, Rivaroxaban 20 mg OD, Edoxaban 60 mg OD) vs. Warfarin				Any NOAC (Dabigatran 110 mg BID, Apixaban 5 mg BID, Rivaroxaban 20 mg OD, Edoxaban 30 mg OD) vs. Warfarin			
	Weighted Average	95% CI Lower	95% CI Upper	p-value	Weighted Average	95% CI Lower	95% CI Upper	p-value
	Effect OR				Effect OR			
Stroke or Systemic Embolism	0.786	0.715	0.864	<0.001	0.909	0.830	0.996	0.041
Stroke	0.801	0.728	0.881	<0.001	0.937	0.855	1.027	0.166
Hemorrhagic stroke	0.497	0.402	0.615	<0.001	0.433	0.346	0.540	<0.001
Ischemic or uncertain type of stroke	0.919	0.825	1.023	0.123	1.128	1.018	1.250	0.021
Systemic Embolism	0.600	0.417	0.863	0.006	0.811	0.578	1.138	0.225
Major Bleeding	0.848	0.791	0.910	<0.001	0.727	0.676	0.783	<0.001
Intracranial Bleeding	0.479	0.405	0.566	<0.001	0.412	0.345	0.493	<0.001
Gastrointestinal Bleeding	1.287	1.150	1.440	<0.001	1.025	0.910	1.155	0.688
Myocardial Infarction	0.945	0.826	1.082	0.413	1.032	0.904	1.178	0.640
Death from any cause	0.904	0.853	0.958	0.001	0.894	0.844	0.948	<0.001

Only endpoints available in all studies are reported. NOAC  =  new oral anticoagulant drug; BID  =  twice daily; OD  =  once daily; CI  =  confidence interval; OR  =  odds ratio.

### Imputed placebo analysis and proportion of warfarin effect preserved

**Figure 2:**
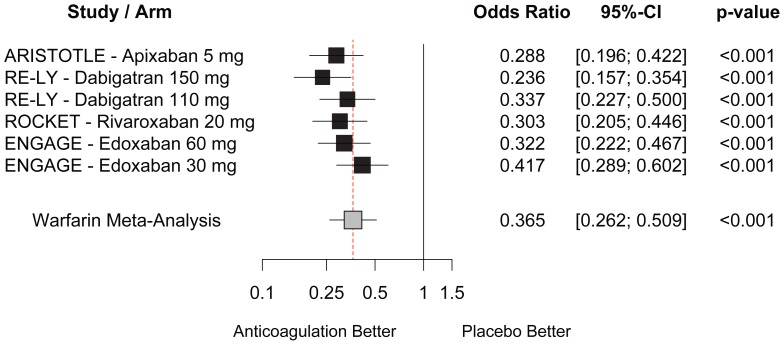
Imputed placebo analysis. Comparison of new oral anticoagulants versus imputed placebo on the risk of stroke.

The comparison of each NOAC versus an imputed placebo on the risk of stroke is shown in figure 2. All NOACs effectively reduced the risk of stroke (all p<0.001). OR ranged between 0.236 for dabigatran 150 mg, and 0.417 for edoxaban 30 mg.

**Figure 3:**
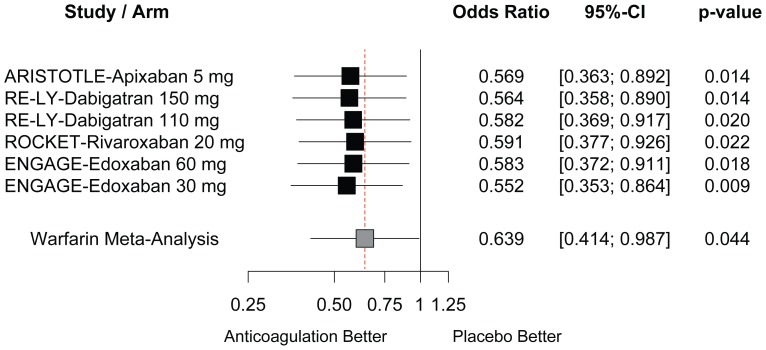
Imputed placebo analysis. Comparison of new oral anticoagulants versus imputed placebo on the risk of all-cause mortality.

All NOACs reduced the risk of all-cause mortality (figure 3) to a similar extent, with ORs ranging between 0.552 and 0.591 (all p<0.05). Overall, risk reductions were somewhat larger with NOACs than with warfarin (OR 0.639, 95% CI: 0.414 to 0.987, p = 0.044) but not formally significant.

**Figure 4:**
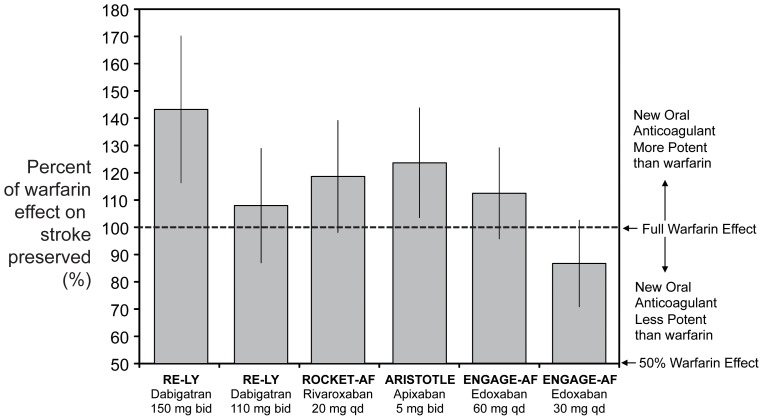
Estimated proportion of warfarin benefit by new oral anticoagulants on the risk of stroke.

**Figure 5:**
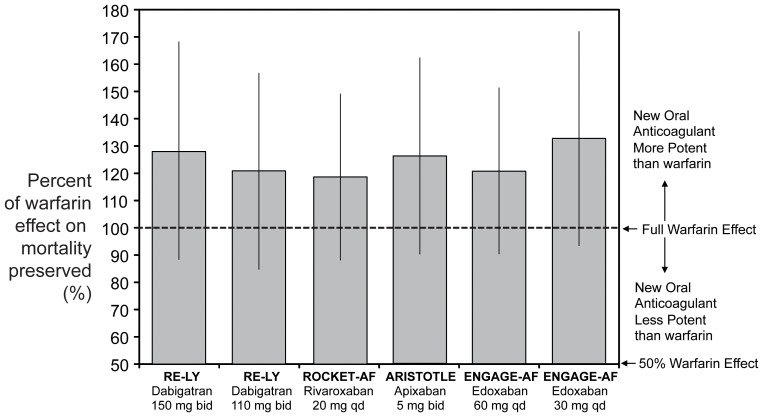
Estimated proportion of warfarin benefit by new oral anticoagulants on the risk of all-cause mortality.

The estimated proportion of warfarin benefit retained on stroke is shown in figure 4. In increasing order, edoxaban 30 mg preserved 87% (95% CI 71–103) of the protective effect of warfarin, followed by dabigatran 110 mg (108%; 95% CI: 87–129), edoxaban 60 mg (112%; 95% CI: 96–129), rivaroxaban (119%; 95% CI: 98–139), apixaban (124%; 95% CI: 103–144) and dabigatran 150 mg (143%; 95% CI: 116–170). The estimated proportion of warfarin benefit retained on all-cause mortality is shown in figure 5. In increasing order, rivaroxaban preserved 118% (95% CI 87–148) of the protective effect of warfarin, followed by dabigatran 110 mg (121%; 95% CI: 85–157), edoxaban 60 mg (121%; 95% CI: 90–151), apixaban (126%; 95% CI: 90–162), dabigatran 150 mg (128%; 95% CI: 87–168) and edoxaban 30 mg (133%; 95% CI 93–172).

### Adjusted indirect comparisons between edoxaban and other agents

OR and 95% CI are reported in tables 3 (efficacy outcomes) and 4 (safety outcomes). The risk of stroke/systemic embolism was significantly higher with edoxaban 30 mg than with dabigatran 150 mg orapixaban. The risk of total stroke and ischemic (or uncertain) stroke was also significantly higher with edoxaban 30 mg than with dabigatran 150 mg, rivaroxaban or apixaban. Apart from systemic embolism and myocardial infarction, which were higher with edoxaban 30 mg than with rivaroxaban, none of the other outcomes showed statistically significant differences between edoxaban 30 mg and any other NOAC. There were no significant differences between the higher dose of edoxaban (60 mg) and any other NOAC in the efficacy outcomes, apart from a slightly higher risk of stroke with edoxaban than rivaroxaban (p = 0.032). The risk of all-cause death did not differ between either dose of edoxaban and other NOACs. The risk of major bleedings (table 4) was significantly lower with edoxaban 30 mg than any other NOAC, and that of gastrointestinal bleedings was lower with edoxaban 30 mg compared with rivaroxaban and both doses of dabigatran. The risk of intracranial bleeding was lower with edoxaban 30 mg versus rivaroxaban. The higher dose of edoxaban did not differ significantly from any other NOAC in terms of safety outcomes, apart from a lower risk of major bleeding compared to rivaroxaban and higher risk of gastrointestinal bleedings compared to apixaban.

**Table 3:** Odds ratio (with 95% confidence interval) of indirect comparisons of edoxaban versus dabigatran, rivaroxaban and apixaban. Efficacy end-points. Significant comparisons are printed in bold.

		Stroke or Systemic Embolism	Stroke	Hemorragic Stroke	Ischemic oruncertain stroke	Systemic Embolism	Myocardial Infarction	All-cause Death
**Edoxaban 30 mg vs.**	*Dabigatran 110 mg bid*	1.210 (0.922–1.589) p = 0.17	1.238 (0.953–1.609) p = 0.109	1.067 (0.514–2.214) p = 0.862	1.279 (0.961–1.702) p = 0.091	1.767 (0.747–4.179) p = 0.195	0.917 (0.628–1.338) p = 0.652	0.95 (0.801–1.126) p = 0.552
	*Dabigatran 150 mg bid*	**1.684 (1.265–2.242) p<0.001**	**1.767 (1.337–2.334) p<0.001**	1.258 (0.588–2.691) p = 0.555	**1.866 (1.378–2.528) p<0.001**	2.060 (0.852–4.983) p = 0.109	0.936 (0.641–1.366) p = 0.731	0.979 (0.826–1.161) p = 0.808
	*Rivaroxaban 20 mg qd*	1.250 (0.977–1.599) p = 0.076	**1.379 (1.072–1.773) p = 0.012**	0.571 (0.308–1.059) p = 0.076	**1.555 (1.172–2.062) p = 0.002**	**5.560 (1.822–16.967) p = 0.003**	**1.503 (1.062–2.127) p = 0.022**	0.935 (0.801–1.093) p = 0.400
	*Apixaban 5 mg qd*	**1.382 (1.068–1.788) p = 0.014**	**1.451 (1.137–1.851) p = 0.003**	0.650 (0.37–1.143) p = 0.134	**1.563 (1.187–2.057) p = 0.001**	1.437 (0.593–3.483) p = 0.422	1.372 (0.954–1.974) p = 0.088	0.971 (0.832–1.134) p = 0.711
**Edoxaban 60 mg vs.**	*Dabigatran 110 mg bid*	0.862 (0.649–1.143) p = 0.302	0.956 (0.732–1.248) p = 0.739	1.747 (0.872–3.501) p = 0.116	0.893 (0.666–1.199) p = 0.452	0.912 (0.36–2.311) p = 0.846	0.718 (0.488–1.055) p = 0.092	1.002 (0.846–1.187) p = 0.984
	*Dabigatran 150 mg bid*	1.199 (0.891–1.612) p = 0.23	**1.363 (1.026–1.811) p = 0.032**	2.060 (0.995–4.262) p = 0.052	1.304 (0.955–1.779) p = 0.095	1.063 (0.411–2.752) p = 0.899	0.733 (0.498–1.078) p = 0.114	1.033 (0.871–1.224) p = 0.711
	*Rivaroxaban 20 mg qd*	0.890 (0.687–1.152) p = 0.375	1.064 (0.822–1.376) p = 0.637	0.935 (0.525–1.664) p = 0.819	1.086 (0.812–1.452) p = 0.578	2.870 (0.891–9.244) p = 0.077	1.176 (0.824–1.679) p = 0.372	0.987 (0.845–1.152) p = 0.864
	*Apixaban 5 mg qd*	0.984 (0.752–1.287) p = 0.905	1.119 (0.872–1.437) p = 0.376	1.065 (0.634–1.787) p = 0.813	1.091 (0.822–1.449) p = 0.545	0.742 (0.286–1.923) p = 0.539	1.074 (0.74–1.558) p = 0.707	1.024 (0.878–1.195) p = 0.761

**Table 4:** Odds ratio (with 95% confidence interval) of indirect comparisons of edoxaban versus dabigatran, rivaroxaban and apixaban.

		Major Bleeding	Intracranial Bleeding	Gastrointestinal Bleeding
**Edoxaban 30 mg vs.**	*Dabigatran 110 mg bid*	**0.581 (0.47–0.719) p<0.001**	1.033 (0.592–1.803) p = 0.909	**0.626 (0.443–0.884) p = 0.008**
	*Dabigatran 150 mg bid*	**0.498 (0.404–0.614) p<0.001**	0.740 (0.441–1.243) p = 0.255	**0.465 (0.334–0.649) p<0.001**
	*Rivaroxaban 20 mg qd*	**0.454 (0.368–0.56) p<0.001**	**0.47 (0.288–0.767) p = 0.003**	**0.421 (0.308–0.575) p<0.001**
	*Apixaban 5 mg qd*	**0.672 (0.544–0.829) p<0.001**	0.729 (0.451–1.177) p = 0.196	0.768 (0.543–1.087) p = 0.137
**Edoxaban 60 mg vs**.	*Dabigatran 110 mg bid*	0.979 (0.802–1.194) p = 0.831	1.539 (0.907–2.611) p = 0.11	1.142 (0.824–1.581) p = 0.426
	*Dabigatran 150 mg bid*	0.839 (0.691–1.02) p = 0.078	1.103 (0.677–1.797)p = 0.695	0.849 (0.621–1.16) p = 0.303
	*Rivaroxaban 20 mg qd*	**0.764 (0.628–0.93) p = 0.007**	0.700 (0.443–1.107) p = 0.127	0.768 (0.575–1.026) p = 0.074
	*Apixaban 5 mg qd*	1.131 (0.929–1.377) p = 0.22	1.086 (0.695–1.697) p = 0.718	**1.400 (1.009–1.944) p = 0.044**

Safety end-points. Significant comparisons are printed in bold.

## Discussion

The main novel finding of the present study is the estimate, obtained through an imputed placebo analysis, of the proportion of warfarin effect preserved by all NOACs on stroke and all-cause mortality in patients with non valvular AF. We based our estimate on a landmark meta-analysis of randomized trials that compared adjusted-dose warfarin versus placebo [1] and four pivotal non-inferiority trials in which 71,683 patients were randomized to adjusted-dose warfarin or NOACs [3]–[6].

### Imputed placebo analysis

This kind of analysis is increasingly performed to estimate how might be the effect of a new treatment if compared versus placebo in the case that a placebo-controlled trial with the new agent would be unethical or unfeasible. Although there is always concern about the value of historic control data, imputed placebo analyses are required by drug regulatory Agencies. For example, the Food and Drug Administration (FDA) approved the use of enoxaparin in the treatment of acute coronary syndrome on the basis of an imputed placebo analysis that included a meta-analysis of randomized trials of unfractionated heparin plus aspirin versus aspirin alone [44], and one randomized comparison of enoxaparin versus unfractionated heparin [45]. Crucial for FDA approval was the demonstration of the high probability that enoxaparin retained at least 80% of the therapeutic effect of unfractionated heparin [23].

In the case of NOACs, placebo controlled trials in patients with non valvular AF would be unethical because warfarin is highly effective in preventing stroke in these patients [1]. When conducting an imputed placebo analysis, two main conditions are required: (a) there is unequivocal historical evidence, that may or may not be obtained through a meta-analysis, of the comparator's superior efficacy versus placebo; (b) the patients enrolled in the trials of active comparator versus placebo and new treatment versus active comparator share common clinical features.

In the present analysis, all NOACs significantly lowered the risk of stroke versus imputed placebo, with reductions ranging between 71% with the higher dose of dabigatran and 38% with the lower dose of edoxaban (all p<0.001). Consequently, all NOACs retained more that 100% of the benefit of warfarin with the exception of edoxaban 30% that, however, retained 87% of its benefit. The higher dose of dabigatran, apixaban and the lower dose of edoxaban were the sole NOACs that significantly reduces all-cause mortality versus imputed placebo.

Our findings confirmed the results of a recent meta-analysis [46] in showing that NOACs, as a whole, are superior to warfarin in reducing the primary composite outcome of stroke/systemic embolism and the secondary outcomes of death and hemorrhagic stroke. While intracranial bleedings were less frequent with NOACs than warfarin, gastrointestinal bleedings were more frequent with NOACs, but only with the higher dose regimens.

In the present analysis we focused on edoxaban as the latest entry in the available scenario of NOACs. In the ENGAGE-AF TIMI 48 trial [6], edoxaban 30 mg was non-inferior to adjusted dose warfarin on the primary composite outcome of stroke/systemic embolism and reduced by 13% the risk of all-cause death (p = 0.006) and by 15% the risk of cardiovascular death (p = 0.008). Also, the composite of death or disabling stroke was by 10% lower (p = 0.02) with edoxaban 30 mg than it was with warfarin. In the ENGAGE AF-TIMI 48 trial, edoxaban 30 mg was also associated with a 53% lower risk of major bleeding, and a 33% lower risk of gastrointestinal bleedings versus warfarin.

Skjøth and coworkers recently published an indirect comparison analysis between different NOACs, including edoxaban [47]. Such analysis, however, did not estimate the benefits of each agent versus imputed placebo and the proportion of the warfarin effect preserved. The present study and that by Skjøth and coworkers share the conclusion that edoxaban 60 mg is comparable to apixaban, rivaroxaban and the lower dose of dabigatran, but inferior to the higher dose of dabigatran, for prevention of stroke. In terms of bleeding end-points, the higher dose of edoxaban is comparable to both doses of dabigatran, and associated with less major bleedings than rivaroxaban and more gastrointestinal bleedings than apixaban [47]. Conversely, the lower dose of edoxaban is comparable to the lower dose of dabigatran, but inferior to all other NOACs for prevention of stroke. The poorer efficacy of the lower dose of edoxaban appears to be outweighed by a higher safety, as reflected by a less risk of major bleedings versus all other NOACs and a less risk of gastrointestinal bleedings versus rivaroxaban and both doses of dabigatran.

Our study extends the conclusions by Skjøth and coworkers in showing that, despite its less antithrombotic efficacy, the lower dose of edoxaban significantly reduces the risk of any stroke (by 58%) and all-cause mortality (by 30%) when compared with a putative placebo. At the point estimate, the lower dose of edoxaban preserved 87% of the benefit of warfarin on stroke and 133% of the benefit of warfarin on all-cause mortality. Notably, the 95% CI of the estimated proportion of the warfarin benefit on stroke preserved by edoxaban 30 mg ranged between 69% in the worst case (i.e., the lower limit of the 95% CI) and 103% in the best case (i.e., the upper limit of the 95% CI). For all-cause mortality, it ranged between 93% in the worst case and 172% in the best case.

The preservation of a pre-specified fraction of the benefit of the control drug by the test drug is a concept that is applied routinely in non-inferiority trials [30]. FDA suggests that non-inferiority trials can be considered statistically persuasive when the test drug preserves at least 60% of the effect of the control treatment [48]. Thus, both doses of edoxaban were significantly more effective than imputed placebo in reducing the risk of stroke and preserved a substantial proportion of the benefit of warfarin, in line with the FDA guidance [48].

### Limitations of the study

The indirect comparison analysis is used to estimate efficacy or safety differences between treatments in the absence of direct head-to-head comparisons [39], [40]. It is unlikely that direct comparisons between different NOACs will be ever undertaken. However, the indirect comparison analysis has well recognized inherent limitations [16], [17]. It assumes that the differences tested in the analysis between any NOAC and the common comparator (warfarin in our case) would have been similar (‘similarity assumption’) also in the context of a different trial population exposed to a different NOAC. The stability of relative treatment effects across trials would make warfarin a credible common comparator. The methods assumes, for example, that the efficacy and safety differences between dabigatran and warfarin found in the RE-LY [3] study would have been the same in the context of the patient population and trial methodology of ROCKET AF [4] or ENGAGE-AF TIMI 48 [6]. By contrast, some differences exist between the four major trials versus warfarin [3]–[6] that could limit the validity of the similarity assumption by making unclear whether the different effects versus warfarin would be attributable to the NOAC alone. Of utmost importance, the risk of thromboembolic complications, reflected by the CHADS_2_ score, was higher in the ROCKET AF [4] and ENGAGE AF-TIMI 48 [6] than in the other trials (table 1). However, none of the subgroups analyses of any NOAC versus warfarin on the primary outcome was statistically significant for interaction by CHADS_2_ score [3]–[6]. Other confounding factors that may limit the validity of indirect comparison analysis in our setting include the open (RE-LY [3]) versus double blind (other trials) design of warfarin administration, the average time in therapeutic range and the concomitant use of aspirin and other drugs.

## Conclusions

In the present study, we tried to put the results of ENGAGE AF-TIMI 48 trial [6] in the scenario of available outcome data on NOACs. Notwithstanding the known caveats of indirect comparisons, while the higher dose of edoxaban did not show important differences from other NOACs in terms of efficacy and safety, the 30 mg dose showed some distinctive features. The better safety profile in terms of major bleedings compared to all other NOACs, and of gastrointestinal bleedings compared to dabigatran and rivaroxaban, would make the lower dose of edoxaban a reasonable option in patients with high or very high risk of bleeding [36]. The lower relative antithrombotic efficacy versus all other NOACs, except the lower dose of dabigatran, should be considered in the light of two findings: 1) the reduction of all-cause mortality in the head-to-head comparison versus warfarin; 2) the significant protective effect on stroke and all-cause mortality in the imputed-placebo analysis and the preservation of a substantial proportion of the protective benefit of warfarin on both outcome measures.

## Supporting Information

PRISMA Checklist S1 PRISMA (Preferred Reporting Items for Systematic reviews and Meta-Analyses) statement for reporting systematic reviews and meta-analyses.
(DOCX)

Protocol S2 Protocol and analysis plan.
(DOCX)
