# Supplementary material for: Edoxaban in the Evolving Scenario of Non Vitamin K Antagonist Oral Anticoagulants Imputed Placebo Analysis and Multiple Treatment Comparisons
Source: PLoS One. 2014 Jun 23;9(6):e100478. doi: 10.1371/journal.pone.0100478 (PMC4067355; doi:10.1371/journal.pone.0100478)
Supplement: Protocol S2 — Protocol and analysis plan. (DOCX) [file pone.0100478.s002.docx]

**Protocol (Supplemental Text S2)**

**Study protocol for the systematic review and updated meta-analysis of the efficacy and safety of edoxaban in relation to other new oral anticoagulant in non-valvular atrial fibrillation by indirect treatment comparisons and imputed placebo analysis**

**Background & rationale**

Vitamin K antagonists (VKA) have long been the only oral anticoagulant agents available for effective thromboprophylaxis in patients with atrial fibrillation (AF). In a landmark meta-analysis of trials conducted in AF patients randomized to either adjusted-dose warfarin versus placebo, active treatment reduced the risk of stroke by 64% (95% confidence interval (CI): 49% to 74%), ischemic stroke by 67% (CI: 54% to 77%), and all-cause mortality by 26 % (CI 3% to 43%) (1).

Such benefit made it unethical to compare new oral anticoagulant agent (NOAC) with placebo in subsequent RCTs. Consequently, the major studies published over the past few years with the direct thrombin inhibitor dabigatran and the factor Xa inhibitors rivaroxaban, apixaban, and, lastly, edoxaban, were active controlled non-inferiority trials versus adjusted-dose warfarin (2).

The findings of recent pivotal non-inferiority phase III randomized trials lead to the marketing approval of dabigatran, apixaban and rivaroxaban (3-5) by regulatory agencies.

More recently the results of the “Effective Anticoagulation with Factor Xa Next Generation in Atrial Fibrillation–Thrombolysis in Myocardial Infarction 48 (ENGAGE AF-TIMI 48)” trial (6), expanded NOACs scenario in patients with non valvular AF. Although this latter compound has not gained a marketing approval to date, it is likely that in the near future it will be made available to treat patients, thus requiring further and careful scrutiny before an evidence based choice could be made.

Taken together NOACs are valuable alternative to warfarin (7, 8), however the physician has few arguments to direct his/her choice to one over the other in the absence of direct head-to-head comparisons. Moreover, it is still not known on a quantitative basis how much of the warfarin effect is retained by each NOAC on selected outcomes including stroke and mortality. The preservation of a pre-specified fraction of the benefit of the control drug by the experimental drug is a concept that is applied routinely in non-inferiority trials (9). In a guidance document, the FDA suggests that non-inferiority trials can be considered statistically persuasive when the test drug preserves at least 60% of the effect of the control treatment (10). Finally, as new evidence become available it might be useful to know how the experimental therapy would have performed if it had been compared directly to placebo (11, 12). Such comparison requires key elements: the observed effectiveness of experimental treatment compared to the active-control, and the estimated effectiveness of the active-control therapy versus placebo obtained from a meta-analysis.

**Review objectives**

The review has three goals:

- Update the previous estimates of efficacy and safety of NOACs as a class versus adjusted dose warfarin;
- Estimate, through indirect treatment comparisons, the efficacy and safety of edoxaban versus different NOACs;
- Estimate the proportion of warfarin effect retained by NOACs and a the benefit versus a putative placebo on the risk of stroke and all-cause mortality.

**Searches**

Electronic searches will be conducted using the following major medical databases: MEDLINE, ISI Web of Knowledge, SCOPUS without language or date restrictions. Search strategies designed for the MEDLINE scoping searches will be adapted to run on other databases. In addition, information on studies will be sought from publicly accessible trial registers (e.g. [www.clinicaltrials.gov](http://www.clinicaltrials.gov)), and Regulatory Authorities websites.

Search terms and keywords:

1. apixaban [Supplementary Concept] OR apixaban [TW]
2. dabigatran [Supplementary Concept] OR dabigatran [TW]
3. edoxaban [Supplementary Concept] OR edoxaban [TW]
4. rivaroxaban [Supplementary Concept] OR rivaroxaban [TW]
5. atrial fibrillation [MeSH Terms]
6. (clinical[Title/Abstract] AND trial[Title/Abstract]) OR
7. clinical trials[MeSH Terms] OR clinical trial[Publication Type] OR random*[Title/Abstract] OR random allocation[MeSH Terms] OR therapeutic use[MeSH Subheading]

**Types of study to be included**

Non-inferiority Phase III Randomized clinical trials.

**Condition or domain being studied**

Atrial fibrillation (AF).

**Interventions**

Novel oral anticoagulants: dabigatran rivaroxaban, apixaban, and edoxaban.

**Comparator / Active control**

Adjusted dose warfarin.

**Outcomes**

**Efficacy outcomes:** composite of stroke and systemic embolism (i.e., the primary efficacy outcome event in each of the four trials), stroke (i.e., all strokes), hemorrhagic stroke, ischemic or uncertain type of stroke, systemic embolism, myocardial infarction and all-cause death.

**Safety outcomes:** major bleeding, intracranial bleeding, gastrointestinal bleeding.

**Data extraction**

Data extraction forms will be developed using Microsoft Access. Data will be extracted by one reviewer and checked by a second. Disagreements will be resolved through consensus, or referral to a third reviewer if disagreements will persist. Data will be extracted on the following: study details (identifier, study design, location, year, length of follow up, industry sponsorship), participant details (number of participants, age, gender), intervention details (drug name, dose, timing, etc.), and comparator intervention details. Data from all arms of multi-arm trials will be extracted. Dichotomous data will be extracted as number of events in intervention and control groups and numbers of participants, following an intention to treat (ITT) approach.

**Quality assessment**

Studies will be assessed using the Cochrane Collaboration’s Risk of Bias Tool. This is used to assign a rating of high, low or unclear risk of bias for the following domains: selection bias, performance bias, detection bias, attrition bias, and reporting bias. Summary assessments of risk of bias (high, low or unclear) will be derived for each outcome in each trial. Assessments will be carried out by one reviewer and checked by a second. Disagreements will be resolved through consensus or referral to a third reviewer where necessary.

**Strategy for data synthesis and analysis plan**

Evidence syntheses will include:

- The average expected effect of NOACs for both efficacy and safety outcomes as a class versus warfarin using the inverse of the variance of the log(odds ratio (OR)) as weights.
- Multiple treatment comparisons between edoxaban and other NOACs using the Bucher method (i.e. adjusted indirect comparisons) with warfarin as common comparator.
- Estimates of the effect of NOACs versus an imputed placebo and the proportion of the warfarin treatment effect retained by each NOAC. For this purpose, the warfarin treatment effect will be derived from a random-effects meta-analysis of 6 historical placebo-controlled trials using the OR as the analysis metric.

We will present results in tables, forest plots, and bar plots.

**Dissemination plans**

We will disseminate the research results through peer-reviewed journals and through methodological and clinical conferences.

**Contact details for further information**

Paolo Verdecchia

Hospital of Assisi, Department of Medicine

Via Valentin Müller, 1

06081 – Assisi, Italy

Fax: 075 8139301

E-mail: [verdec@tin.it](mailto:verdec@tin.it)

Gianpaolo Reboldi

Department of Medicine

University of Perugia

06132 Perugia, ITALY

**Organisational affiliation of the review**

Hospital of Assisi, Department of Medicine, Assisi, ITALY

University of Perugia School of Medicine, Perugia, ITALY

**Review team**

Dr Paolo Verdecchia, Hospital of Assisi, ITALY

Dr Fabio Angeli, University Hospital, Perugia, ITALY

Dr Gregory YH Lip, University of Birmigham, UK

Dr. Gianpaolo Reboldi, University of Perugia, ITALY

**Start date**

25 November 2013

**Completion date**

31 January 2014

**Funding sources/sponsors**

None

**References**

1. Hart RG, Pearce LA, Aguilar MI. Adjusted-dose warfarin versus aspirin for preventing stroke in patients with atrial fibrillation. Annals of internal medicine. 2007;147(8):590-2.

2. Connolly SJ, Eikelboom J, O'Donnell M, Pogue J, Yusuf S. Challenges of establishing new antithrombotic therapies in atrial fibrillation. Circulation. 2007;116(4):449-55.

3. Connolly SJ, Ezekowitz MD, Yusuf S, Eikelboom J, Oldgren J, Parekh A, et al. Dabigatran versus warfarin in patients with atrial fibrillation. N Engl J Med. 2009;361(12):1139-51.

4. Patel MR, Mahaffey KW, Garg J, Pan G, Singer DE, Hacke W, et al. Rivaroxaban versus warfarin in nonvalvular atrial fibrillation. N Engl J Med. 2011;365(10):883-91.

5. Granger CB, Alexander JH, McMurray JJ, Lopes RD, Hylek EM, Hanna M, et al. Apixaban versus warfarin in patients with atrial fibrillation. N Engl J Med. 2011;365(11):981-92.

6. Giugliano RP, Ruff CT, Braunwald E, Murphy SA, Wiviott SD, Halperin JL, et al. Edoxaban versus warfarin in patients with atrial fibrillation. N Engl J Med. 2013;369(22):2093-104.

7. Camm AJ, Lip GY, De Caterina R, Savelieva I, Atar D, Hohnloser SH, et al. 2012 focused update of the ESC Guidelines for the management of atrial fibrillation: an update of the 2010 ESC Guidelines for the management of atrial fibrillation. Developed with the special contribution of the European Heart Rhythm Association. European heart journal. 2012;33(21):2719-47.

8. You JJ, Singer DE, Howard PA, Lane DA, Eckman MH, Fang MC, et al. Antithrombotic therapy for atrial fibrillation: Antithrombotic Therapy and Prevention of Thrombosis, 9th ed: American College of Chest Physicians Evidence-Based Clinical Practice Guidelines. Chest. 2012;141(2 Suppl):e531S-75S.

9. Snapinn S, Jiang Q. Preservation of effect and the regulatory approval of new treatments on the basis of non-inferiority trials. Statistics in medicine. 2008;27(3):382-91.

10. Food and Drug Administration. Guidance for Industry: Non-Inferiority Clinical Trials2010 2 January 2014. Available from: <http://www.fda.gov/downloads/Drugs/GuidanceComplianceRegulatoryInformation/Guidances/UCM202140.pdf>.

11. Hasselblad V, Kong DF. Statistical methods for comparison to placebo in active-control trials. Drug Info J. 2001;35:435-9.

12. Snapinn S, Jiang Q. Indirect comparisons in the comparative efficacy and non-inferiority settings. Pharmaceutical statistics. 2011;10(5):420-6.
